# A Novel Sensor for Monitoring of Iron(III) Ions Based on Porphyrins

**DOI:** 10.3390/s120608193

**Published:** 2012-06-13

**Authors:** Dana Vlascici, Eugenia Fagadar-Cosma, Iuliana Popa, Vlad Chiriac, Mayte Gil-Agusti

**Affiliations:** 1 Faculty of Chemistry-Biology-Geography, West University of Timisoara, Pestalozzi Street 16, 300115-Timisoara, Romania; E-Mail: danavlascici@yahoo.com; 2 Institute of Chemistry Timisoara of Romanian Academy, M. Viteazul Ave. 24, 300223-Timisoara, Romania; 3 National Institute of Research for Electrochemistry and Condensed Matter, Timisoara, Aurel Paunescu Podeanu Street 144, 300860-Timisoara, Romania; E-Mail: iuliana_p19@yahoo.com; 4 Instituto Tecnológico de la Energía (ITE), Avenida Juan de la Cierva 24, 46980 Paterna, Valencia, Spain; E-Mail: mayte.gil@ite.es

**Keywords:** porphyrins, ion-selective electrode, potentiometry, lithium ion batteries, iron(III), PVC membrane

## Abstract

Three A_3_B porphyrins with mixed carboxy-, phenoxy-, pyridyl-, and dimethoxy-substituent functionalization on the *meso*-phenyl groups were obtained by multicomponent synthesis, fully characterized and used as ionophores for preparing PVC-based membrane sensors selective to iron(III). The membranes have an ionophore:PVC:plasticizer composition ratio of 1:33:66. Sodium tetraphenylborate was used as additive (20 mol% relative to ionophore). The performance characteristics (linear concentration range, slope and selectivity) of the sensors were investigated. The best results were obtained for the membrane based on 5-(4-carboxyphenyl)-10,15,20-tris(4-phenoxyphenyl)-porphyrin plasticized with *bis*(2-ethylhexyl)sebacate, in a linear range from 1 × 10^−7^–1 × 10^−1^ M with a slope of 21.6 mV/decade. The electrode showed high selectivity with respect to alkaline and heavy metal ions and a response time of 20 s. The influence of pH on the sensor response was studied. The sensor was used for a period of six weeks and the utility has been tested for the quantitative determination of Fe(III) in recovered solutions from spent lithium ion batteries and for the quantitative determination of Fe(III) in tap water samples.

## 1. Introduction

Iron is an essential element in biological processes, playing an important role as oxygen carrier, in storage and electron transport. This is the reason why major impacts can appear due to its metabolism deregulation. Thus, an abnormal higher level of iron in the body leads to haemochromatosis and its deficiency leads to anemia. Besides, technical developments regarding Ni, Co and Mn recovery in the recycling process of Li-batteries implies iron monitoring in synthetic leach liquor resulted from reductive leaching. This is the reason why iron must be precisely determined in biological, chemical, environmental [1–12] and industrial samples [13,14].

In order to monitor Fe^3+^ ions from different samples, many techniques have been used. A sequential injection procedure was used for determination of Fe(II) and Fe(III) in aquatic samples by a complex procedure. Fe(II) was determined by complexation with 1,10-phenantroline and Fe(III) analysis was performed after reduction in Jones and copperized cadmium columns. For Fe(III) the quantification limits were 0.05 and 0.1 mg L^−1^, with a sampling frequency of 20 h^−1^ [1]. Rapid and low cost colorimetric spot-test determination of Fe(III) was applicable for concentration of Fe(III) from 6 to 45 mmol·L^−1^ [2]. Separation and preconcentration of Fe(III) ions in various water samples was achieved by sorption at a pH of 3.5 in a minicolumn obtained from Amberlite XAD-4, functionalized with 2,3-dihydroxybenzoic acid by coupling it through an –N=N–spacer, then eluted using H_3_PO_4_. The released amount of Fe^3+^ was determined by flame atomic absorption spectrometry with a recovery of more than 98% [3]. Inductively coupled plasma atomic emission spectrometry was used for determination of Fe (III) in aqueous solutions by modified nanometer device based on SiO_2_ using 5-sulfosalicylic acid as a solid-phase extractant for separation and preconcentration [4].

The use of ion-selective electrodes for iron(III) detection as a fast and low-cost method was also reported in the last years. A sensor based on 2-[(2-hydroxy-1-propenylbuta-1,3-dienylimino)-methyl]-4-*p*-tolylazophenol as carrier for the determination of Fe(III) in the presence of Fe(II), in a concentration range of 3.5 × 10^−6^–4 × 10^−2^ M, with a super Nernstian slope of 28.5 (±0.5) mV/decade and in a pH range from 4.5 to 6.5 has been reported [5]. Mollagh *et al.* [6] have developed PVC membrane and coated wire sensors based on 1-phenyl-3-pyridin-2-ylthiourea with the best performances in the range 3.0 × 10^−7^–1.0 × 10^−2^ M and a slope of 20.2 ± 0.8 mV/decade. An iron(III) ion-selective sensor based on a µ-bis(tridentate) ligand was developed by Gupta and collaborators [7]. The sensor showed a linear potential response in a concentration range 6.3 × 10^−6^–1.0 × 10^−1^ M with a Nernstian slope of 20.0 mV/decade, between pH 3.5 and 5.5. Iron was determined in biological and non-biological samples using a sensor based on 1,1′-(iminobis(methan-1-yl-1-ylidene))dinaphtalen-2-ol as carrier with a concentration range between 1.0 × 10^−7^–1.0 × 10^−1^ M and a slope of 19.9 mV/decade [8]. S-Methyl *N*-(methylcarbamoyloxy)thioacetimidate was used as ionophore [9] in another Fe(III)-selective sensor which works in the range 9.1 × 10^−6^–1.0 × 10^−1^ M with a slope of 21.2 mV/decade. An iron ion sensor based on functionalized ZnO nanorods has been developed [10], which was observed to be linear in the concentration range from 10^−5^ to 10^−2^ M with sensitivity of 70.2 ± 2.81 mV/decade. Using bis-benzilthiocarbohydrazide as sensing material, the autors [11] have reported the fabrication of an iron(III)-selective sensor which works from 1.0 × 10^−7^ to 1.0 × 10^−2^ M with a Nernstian slope in a pH range of 1.6–4.3.

Only one potentiometric sensor for Fe^3+^ ions detection based on porphyrins, namely: 5,10,15,20-tetrakis(pentaflurophenyl)-21H,23H-porphyrin, as membrane carrier was presented [12]. The porphyrin-based sensor revealed good selectivity for Fe^3+^ over a wide variety of other cations at pH 3–4 in the concentration range 1.0 × 10^−4^–1.0 × 10^−6^ M. This method was applied to the direct determination of iron in tap water samples.

**Figure 1.**
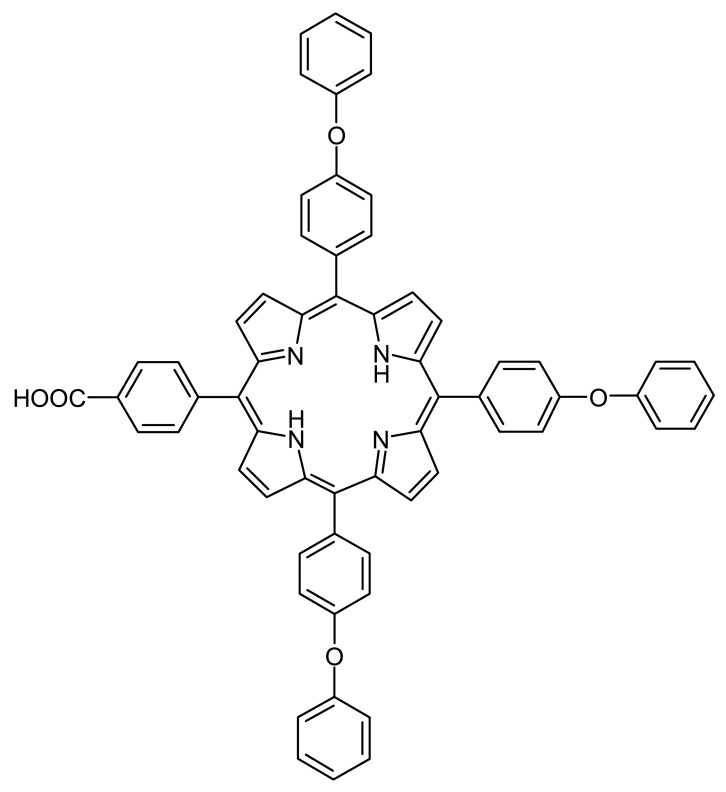
The chemical structure of 5-(4-carboxyphenyl)- 10,15,20-tris(4-phenoxyphenyl)-porphyrin.

In the present paper we have used three A_3_B porphyrins with mixed functionalization to obtain iron(III)-potentiometric sensors. The best results were obtained for the sensor based on a novel synthesized porphyrin structure, 5-(4-carboxyphenyl)-10,15,20-tris(4-phenoxyphenyl)-porphyrin (Figure 1) plasticized with bis(2-ethylhexyl) sebacate. The sensor was used for iron monitoring in synthetic leach liquor resulting from reductive leaching [13] in the recycling process of Li-batteries. The leaching solution from spent battery material contains large quantities of valuable metals Ni, Co and Mn, as well as impurities, Cu and Fe. Recent developments regarding Ni, Co and Mn recovery implies that copper is removed through replacement by iron powder, followed by iron precipitation. The final results show that Cu and Fe can be removed 99 wt. % at the least [14]. For the monitoring of iron removal from leach solution, it was formulated the present new sensor based on porphyrin ionophore.

## 2. Experimental

### 2.1. Reagents

The porphyrin 5-(4-carboxyphenyl)-10,15,20-tris(4-phenoxyphenyl)-porphyrin (**P1**) was synthesized as presented below. The porphyrins 5-(4-pyridyl)-10,15,20-tris(3,4-dimethoxyphenyl)-porphyrin (**P2**) and 5-(4-pyridyl)-10,15,20-tris(4-phenoxyphenyl)-porphyrin (**P3**) were synthesized, purified and characterized in accordance with previously published procedures [15].

For membrane preparation, poly(vinyl)chloride (PVC) high molecular weight, bis(2-ethyl-hexyl)sebacate (DOS), *o*-nitrophenyloctylether (NPOE), dioctylphtalate (DOP), sodium tetraphenylborate (NaTPB) and tetrahydrofuran (THF) were purchased from Fluka and Merck. All salts, acids and base were of analytical reagent grade. Double distilled water was used. The performance of each sensor was investigated by measuring its potential in the concentration range 10^−5^–10^−1^ M of different cationic solutions. In the case of iron(III) the solutions were made in a concentration range up to 10^−7^ M. Stock solutions, 0.1 M, were prepared by dissolving metal chlorides in double distilled water and standardized if necessary. All working solutions were prepared by gradual dilution of the stock solutions.

### 2.2. Apparatus

FT-IR spectra were recorded on a JASCO 430 FT-IR, as KBr pellets, in the 4,000–400 cm^−1^ range. UV-visible spectra were recorded on a Perkin Elmer LAMBDA 12 UV/VIS spectrometer and on a JASCO UV-visible spectrometer, model V-650. Absorption spectra were recorded at ambient temperature using 1 cm path length cells. ^1^H-NMR spectra were obtained on Bruker Avance DRX 400 equipment, using CDCl_3_ as solvent. The HPLC analysis were performed on a JASCO apparatus equipped with a silica gel KROMASIL 100 SIL 5 µm 250 × 4.0 mm column and a MD 1510 detector, at ambient temperature, using UV detection at 419 nm. For MS analysis of porphyrin base an Electrospray Ionization Bruker Esquire 6000 mass spectrometer was used. Thin-layer chromatography (TLC) was performed on silica gel plate 60 F_254_ pre-coated aluminum sheets from Merck.

### 2.3. Ionophore Synthesis and Characterization

The most used direct route to obtain a carboxylic acid porphyrin derivative is through hydrolysis of ester-type porphyrin derivatives, usually ester type Zn-metalloporphyrins [16]. In the reported study, the synthesis of unsymmetrical mixed substituted 5-(4-carboxyphenyl)-10,15,20-tris(4-phenoxyphenyl)-porphyrin was based on modified previous literature data [17,18] condensing a mixture of pyrrole and two appropriately substituted benzaldehydes in a particular ratio, as follows: to a solution consisting of 4-carboxymethylbenzaldehyde (1.58 g, 9.69 mmol) and 4-phenoxybenzaldehyde (5 mL, 29.08 mmol) dissolved in propionic acid (284 mL) as solvent, propionic anhydride (4.97 mL, 38.77 mmol) was added. The mixture was brought to reflux under vigorous stirring. For 25 min a solution of pyrrole (2.689 mL, 38.77 mmol) in propionic acid (6.45 mL) was continuously added to this mixture. The reflux is maintained for 3.5 h. The products were cooled to room temperature and a violet precipitate is formed. The solid is several times washed with hot water, and then solved again in THF over anhydrous Na_2_SO_4_ to remove the humidity. To the new filtered solution, 100 mL hexane were added and filtered next day. The methyl ester was hydrolyzed in basic condition using KOH in a mixture MeOH/water, followed by neutralization with dilute HCl [19]. The solid was separated on reversed phase analytical column chromatography 250 × 4.0 mm, KROMASIL 100, Sil, 5 µm in a mixture of solvents: ethyl acetate-DMSO-propionic acid = 90:10:300 µL. The retention time for the 5-(4-carboxyphenyl)-10,15,20-tris(4-phenoxyphenyl)-porphyrin was 2.69 min, being the major peak, eluted second. TLC analysis on a silica gel plate 60 F_254_ gave R_f_ = 0.38 (EtOAc-hexane, 1:3, v/v). *5-(4-carboxyphenyl)-10,15,20-tris(4-phenoxyphenyl)-porphyrin* (**P1**): dark violet crystals; yield: 7.3%; mp over 300 °C; FT-IR (ν, cm^−1^): 746 and 796 (γ C-H_pyrrole_), 861, 964, 1,234 (aromatic C–O–C), 1,483 (C–O–C and/or ν C=N), 1,587(ν C=C_Ph_), 1,686 (ν C=O), 3,032.5 (ν C-H_Ph_), 3,311(ν N-H); ^1^H-NMR (CDCl_3_), δ, ppm: 8.92–8.94 (d, 6H, β-pyrrole) 8.84 (bs, 2H, β-pyrrole), 8.53 (d, 4H, H-2,6 phenyl) 8.17–8.19 (d, 4H, H-2,6 phenyl), 7.50–7.59 (m, 8H, H-3,5 phenyl), 7.35–7.41 (m, 15H, H-phenyl), 2.70 (br s, 2H, internal-NH-pyrrole); UV-vis, THF (λmax (log ε): 418.48 (5.22); 515.17 (4.53); 550.50 (4.32); 592.56 (4.11); 650.69 (4.04); HPLC R_T_, min: 2.69; TLC (silica gel 60 Å, indicator F_254_, EtOAc-hexane, 1:3, v/v), R_f_: 0.38; MS (ESI^+^): *m/z* = 935.2 M]^+^ (C_63_H_42_N_4_O_5_]^+^·molecular ion, calcd. for M]^+^ 935.03 g/mol; in the ^1^H-NMR spectrum of the methylic ester, a singlet signal located at 4.06 ppm, corresponding to the three protons of -OCH_3_ group is present.

### 2.4. Electrode Membrane Preparation and Measurements

The membranes have an ionophore-PVC-plasticizer in the ratio 1:33:66 composition. Sodium tetraphenylborate was used as additive (20 mol % relative to ionophore). The electroactive material and the solvent mediator were mixed together, and then the PVC and the appropriate amount of THF were added and mixed to obtain a transparent solution. This solution was transferred onto a glass plate of 20 cm^2^, and the THF was allowed to evaporate at room temperature leaving a tough, flexible membrane embedded in a PVC matrix. An 8 mm diameter piece of membrane was cut out and assembled on the Fluka electrode body. The measurements were carried out at room temperature using a Hanna Instruments HI223 pH/mV-meter by setting up the following cell:

$$\text{Ag}|\text{Agcl}|\text{KCl}\;\left(\text{sat}\right)|\text{sample}|\text{ion‐selective membrance}|1.0\times 10^{-2}\text{M}\;{\text{Fe}}^{3+}|\text{Agcl},\text{Ag}$$

Prior to EMF measurements, all the sensors were conditioned for 24 h by soaking in 0.01M FeCl_3_. Potentiometric selectivity coefficients were determined according to the separate solution method by using the experimental EMF values obtained for 0.01 M of the tested cations and a theoretical slope of 19.7 mV/decade of activity for iron(III) cation. The detection limit of each sensor was established at the point of intersection of the extrapolated linear mid-range and final low concentration level segments of the calibration plot.

### 2.5. Analytical Applications

#### 2.5.1. Determination of Iron in Synthetic Leach Liquors from Spent Lithium Ion Batteries

Synthetic leach liquors from spent lithium ion batteries, similar to the real ones [20], containing 1.36 × 10^−2^ M Mn^2+^, 8,48 × 10^−2^M Ni^2+^, 1 M Li^+^, 3.56 × 10^−2^ M Fe^3+^, 0,17 M Co^2+^ and 4,7 × 10^−2^ M Cu^2+^ were prepared by weighing the appropriate amount of each salt in double distillated water. The best obtained iron(III)-sensor was used for monitoring the iron in solutions by direct potentiometry. We have made three tests. First, solution **A** containing just the above mentioned quantities of the cations Mn^2+^, Ni^2+^, Li^+^ and Fe^3+^. Solution **B** was prepared from solution A by adding Co^2+^ and solution **C** from B plus Cu^2+^. The potential of the solutions was measured to see the iron recovery.

#### 2.5.2. Determination of Iron in Tap Water

The sensor was also used for the detection of iron in three samples of tap water by direct potentiometry using a calibration graph.

## 3. Results and Discussion

### 3.1. Response Characteristics of the Electrodes

Many porphyrins [12,21,22] and metalloporphyrins were used in the last years as ionophores for the developing of new polymeric membrane ion-selective electrodes. As a continuation of our previous studies regarding porphyrin based potentiometric sensors [23–26], three porphyrins (P1, P2 and P3) were used as ionophores in the preparation of polymeric membrane sensors.

First, all the membranes were obtained with the same composition, using NPOE as plasticizer and NaTPB as anion excluder. The membranes were tested as iron sensors and against a number of monovalent and bivalent cations. All the porphyrins show selectivity for iron(III). As it results from Table 1, for porphyrins P2 and P3 the results were weak from the working concentration range point of view, and also with super Nernstian values of the slopes. Porphyrin P1 plasticized with NPOE has a better working concentration range, but also a super Nernstian slope value.

It is known that the plasticizers have a strong influence on the potentiometric response of the sensors, and also on the value of the slope. Hoping for an improvement of the slope value, we have made sensors 4 and 5 by changing the plasticizer NPOE with big dielectric constant (ε ≈ 24) for plasticizers having lower dielectric constants: DOS (ε ≈ 4) and DOP (ε ≈ 5.1).

**Figure 2.**
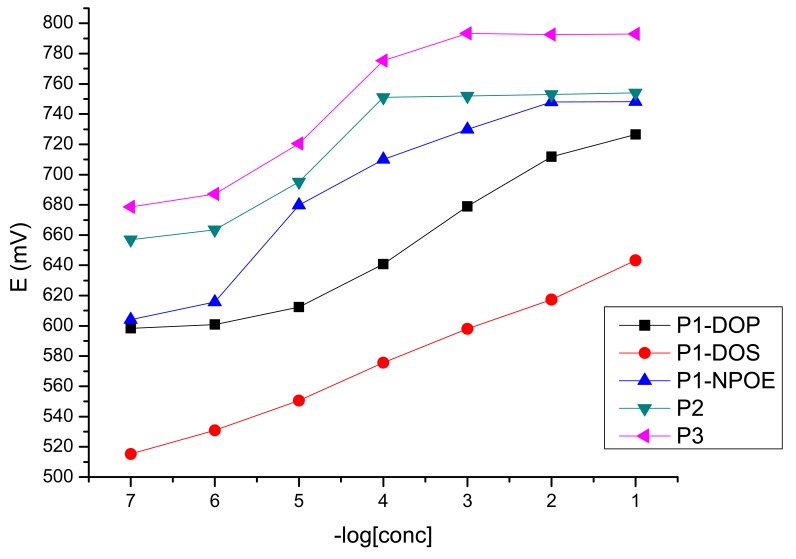
Potentiometric response toward iron(III) of the obtained sensors.

**Figure 3.**
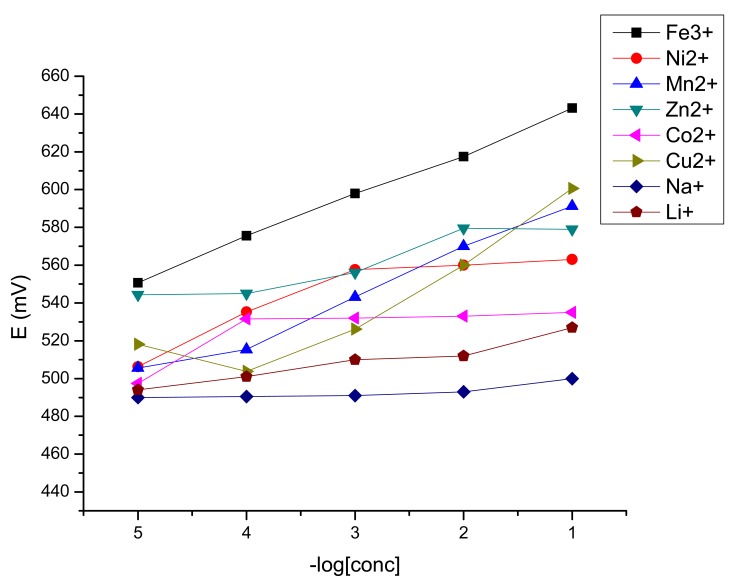
Potentiometric response of porphyrin **P1**-based sensors having the optimum composition of the membrane toward different metal ions.

The potentiometric response toward iron(III) of the obtained sensors is presented in Figure 2. As it could be seen, both from Figure 2 and Table 1, the best results were obtained for the sensor plasticized with DOS, which has a working concentration range from 1 × 10^−1^–1 × 10^−7^ M with a near Nernstian slope of 21.6 mV/decade of activity (y = −21.575x + 662.16, R^2^ = 0.9966). For this sensor, the potentiometric answer toward the tested cations is presented in Figure 3.

The stability and reproducibility of the best obtained sensor were also tested. The standard deviation of 15 replicate measurements made for the 1 × 10^−3^ M solution was ±0.5 mV. The sensor was used for a period of six weeks without significant changes of the potentials.

### 3.2. Detection Limit and Response Time

The practical response time of the sensor to reach 95% of the equilibrium potential was obtained after successive immersion of the electrode in a series of iron(III) ion solutions, each having a 10-fold difference in concentration. The obtained response time was about 20 s as the concentration of iron(III) varies from 10^−4^ to 10^−3^ M. The detection limit for the best performing electrode was established at the point of intersection of the extrapolated linear mid-range and final low concentration level segments of the calibration plot and it is (8.6 ± 0.4) × 10^−8^ M.

### 3.3. Potentiometric Selectivity

The potentiometric selectivity coefficients ( $\log{\text{K}}_{Fe^{3+},X^{+/2+}}^{\text{pot}}$) were calculated by the separate solution method [28] for primary and interfering cations concentration of 1 × 10^−2^ M and are presented in Table 2.

As it could be seen from Table 2, the sensor has very good values of the selectivity coefficients, especially toward Co^2+^, Ni^2+^ and Li^+^, the main components of the leach liquor from spent lithium ion batteries.

### 3.4. Effect of pH

**Figure 4.**
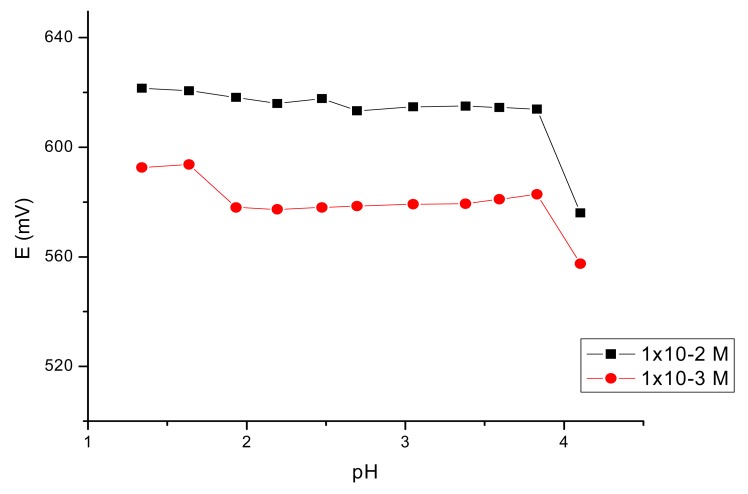
Effect of the pH of the test solution on the potential response of the sensor with best potentiometric answer.

The influence of the pH of the test solutions on the potential response of the electrodes was studied by using the 10^−2^ and 10^−3^ M iron(III) solutions adjusted with HCl and NaOH and the results are given in Figure 4.

As it can be seen, the sensor may be used in a pH range from 2.0–3.8. Above this value of the pH the precipitation of iron(III) hydroxide occurs.

### 3.5. Analytical Applications of the Sensor

As it was mentioned before, the sensor was used for monitoring of iron(III) cation in synthetic solutions from spent lithium ion batteries. The results are presented in Table 3, each of them obtained from four measurements (the standard deviation is given).

We have also tested the sensor for the determination of iron in three samples of tap water. The results, obtained for five measurements are comparatively presented with those of AAS in Table 4.

A good recovery of the amount of iron (III) in the samples was obtained by using the novel sensor, both at lower and higher concentrations, as it results from Tables 3 and 4.

## 4. Conclusions

A novel iron(III)-potentiometric sensor based on a novel structure, namely 5-(4-carboxyphenyl)-10,15,20-tris(4-phenoxyphenyl)-porphyrin, plasticized with bis(2-ethylhexyl)sebacate, in a linear range from 1 × 10^−7^–1 × 10^−1^ M with a slope of 21.6 mV/decade was developed. The electrode has a response time of 20 s; works in a pH range from 2.0–3.8 and has a good selectivity towards a lot of cations. The sensor was used for a period of six weeks and the utility has been tested for the quantitative determination of Fe(III) in recovered solutions from spent lithium ion batteries.

## Figures and Tables

**Table 1.** Composition and response characteristics of the obtained membranes.

**Sensor no.**	**% Composition (w/w) of the membranes**							**Working concentration range (M)**	**Slope (mV/decade)** ^**^

	**Ionophore**			**PVC**	**NPOE**	**DOS**	**DOP**		

	**P1**	**P2**	**P3**						
1	1			33	66			1 × 10^−7^–1 × 10^−2^	31.2 ± 1.0
2		1		33	66			1 × 10^−6^–1 × 10^−4^	43.7 ± 1.5
3			1	33	66			5 × 10^−7^–1 × 10^−4^	41.6 ± 1.3
4	1			33		66		1 × 10^−1^–1 × 10^−7^	21.6 ± 0.7
5	1			33			66	1 × 10^−6^–5 × 10^−2^	28.8 ± 0.8

* All the membranes contain 20 mol. % NaTPB (relative to the ionophore);

** Standard deviation [27].

**Table 2.** Selectivity coefficients of the optimal composition membrane sensor.

**Interfering cation X^+/2+^**	$\log{\text{K}}_{Fe^{3+},X^{+/2+}}^{\text{pot}}$
Fe^3+^	0,00
Ni^2+^	−3,90
Mn^2+^	−1,40
Zn^2+^	−3,45
Co^2+^	−4,00
Cu^2+^	−2,01
Na^+^	−2,50
Li^+^	−4,28

**Table 3.** Analytical application of the iron(III)-sensor in synthetic leach liquor.

**Sample**	**Iron in solution by AAS (g/L)**	**Iron found by the electrode (g/L)**	**Recovery (%)**
**A**		1.97 ± 0.02	99.0
**B**	1.99 ± 0.01	1.95 ± 0.04	98.0
**C**		1.93 ± 0.03	97.0

**Table 4.** Analytical application of the iron(III)-sensor in tap water.

**Sample**	**Iron in water by AAS (ppm)**	**Iron found by the electrode (ppm)**	**Recovery (%)**
**A**	5.30 ± 0.02	5.15 ± 0.10	97.2
**B**	2.85 ± 0.01	2.90 ± 0.08	101.7
**C**	7.53 ± 0.05	7.35 ± 0.12	97.6
